# Distant Metastasis Pattern and Prognostic Prediction Model of Colorectal Cancer Patients Based on Big Data Mining

**DOI:** 10.3389/fonc.2022.878805

**Published:** 2022-04-22

**Authors:** Chuan Liu, Ting Wang, Jiahui Yang, Jixiang Zhang, Shuchun Wei, Yingyun Guo, Rong Yu, Zongbiao Tan, Shuo Wang, Weiguo Dong

**Affiliations:** ^1^ Department of Gastroenterology, Renmin Hospital of Wuhan University, Wuhan, China; ^2^ Department of Geriatric, West China Hospital of Sichuan University, Chengdu, China

**Keywords:** colorectal cancer, distant metastasis, Surveillance Epidemiology and End Results (SEER), nomogram, prognosis

## Abstract

**Aims:**

This study aimed to investigate the distant metastasis pattern from newly diagnosed colorectal cancer (CRC) and also construct and validate a prognostic nomogram to predict both overall survival (OS) and cancer-specific survival (CSS) of CRC patients with distant metastases.

**Methods:**

Primary CRC patients who were initially diagnosed from 2010 to 2016 in the SEER database were included in the analysis. The independent risk factors affecting the OS, CSS, all-cause mortality, and CRC-specific mortality of the patients were screened by the Cox regression and Fine–Gray competitive risk model. The nomogram models were constructed to predict the OS and CSS of the patients. The reliability and accuracy of the prediction model were evaluated by consistency index (C-index) and calibration curve. The gene chip GSE41258 was downloaded from the GEO database, and differentially expressed genes (DEGs) were screened by the GEO2R online tool (*p* < 0.05, |logFC|>1.5). The Kyoto Encyclopedia of Genes and Genomes (KEGG) Pathway and Gene Ontology (GO) annotation and String website were used for enrichment analysis and protein–protein interaction (PPI) analysis of DEGs, respectively, and Cytoscape software was used to construct PPI network and screen function modules and hub genes.

**Results:**

A total of 57,835 CRC patients, including 47,823 without distant metastases and 10,012 (17.31%) with metastases, were identified. Older age, unmarried status, poorly differentiated or undifferentiated grade, right colon site, larger tumor size, N2 stage, more metastatic sites, and elevated carcinoembryonic antigen (CEA) might lead to poorer prognosis (all *p* < 0.01). The independent risk factors of OS and CSS were included to construct a prognosis prediction model for predicting OS and CSS in CRC patients with distant metastasis. C-index and calibration curve of the training group and validation group showed that the models had acceptable predictive performance and high calibration degree. Furthermore, by comparing CRC tissues with and without liver metastasis, 158 DEGs and top 10 hub genes were screened. Hub genes were mainly concentrated in liver function and coagulation function.

**Conclusion:**

The big data in the public database were counted and transformed into a prognostic evaluation tool that could be applied to the clinic, which has certain clinical significance for the formulation of the treatment plan and prognostic evaluation of CRC patients with distant metastasis.

## 1 Introduction

Colorectal cancer (CRC) is the third most common cancer in men and the second in women worldwide (1) and is the second most common cause of cancer-related death in the United States (2). About 1.8 million new cases of CRC occur each year and cause about 860,000 deaths (3). The main cause for the high mortality is metastases in CRC patients (4). It was reported that almost 50% of CRC patients presented with metastatic disease development, and approximately 25% of patients presented with distant metastatic disease at initial diagnosis (5). In CRC patients with distant metastases, the survival rate of patients is dramatically reduced. Therefore, it is necessary to research the diagnosis, treatment, and prognostic factors of CRC in order to improve the survival rate of these patients. There has been a relative insufficiency of literature specific to the subject, including only a few retrospective studies with a small sample size.

Surveillance, Epidemiology, and End Results (SEER) Program (http://seer.cancer.gov/) from the National Cancer Institute (NCI) is one of the largest publicly available cancer datasets worldwide. The SEER program covers approximately 30% of the American population in different geographic regions since 1973 (6). A nomogram is a model that predicts the probability of a patient’s clinical events based on multivariate regression analysis, which can quickly and intuitively predict the prognosis of patients, and is widely used in tumor-related research. But researchers have not yet constructed a prognostic model for CRC with distant metastasis. Therefore, on the one hand, this study used clinical data related to such patients in the SEER database for statistical analysis to describe the characteristics for newly diagnosed CRC patients with distant metastases and analyze distant metastasis patterns systematically. On the other hand, this paper screened out the factors significantly related to prognosis and drew a prognostic prediction model according to this, so as to show the influence degree of each factor on the prognosis of CRC patients with distant metastasis and then predict the overall prognosis of patients. Furthermore, to further research the mechanism of distant metastasis of CRC, bioinformatics methods were also used to screen out gene chips related to CRC distant metastasis from Gene Expression Omnibus (GEO) database, find differentially expressed genes (DEGs), conduct enrichment analysis on them, and screen out hub genes, so as to provide theoretical support for further exploration of pathogenesis and therapeutic targets.

## 2 Materials and Methods

### 2.1 Database and Study Population

Patients with CRC were identified in the SEER database. Due to the unavailability of CRC metastasis details prior to 2010, relevant information of CRC patients who were initially diagnosed between 2010 and 2016 only was collected. According to the third edition of the International Classification of Diseases for Oncology (ICD-O-3), the tumor site was restricted as rectal cancer (C199 and C209) and colon cancer (C180–C189). The following individuals were included: 1) cases of primary CRC with a microscopically confirmed diagnosis and 2) tumor histology based on ICD-O-3 codes 8140, 8210, 8261, and 8263 for adenocarcinoma; 8480 and 8490 for mucinous adenocarcinoma (MC); and signet ring cell carcinoma (SRCC). The patients were excluded if they met the exclusion criteria, as follows: 1) tumor was diagnosed solely on autopsy or death certificate; 2) follow-up information or metastasis details were missing (survival months was 0); and 3) patients with multiple primary cancers. Tumor-Node-Metastasis (TNM) stage was defined based on the seventh edition of the American Joint Committee on Cancer (AJCC) TNM staging system (7). The screening flowchart according to these inclusion and exclusion criteria could be seen in **Supplementary Figure 1**.

The following data of indicators were extracted: age at diagnosis, gender, marital status, race/ethnicity, insurance status, histology type, primary tumor site, grade, tumor size, TNM stage, serum carcinoembryonic antigen (CEA) level, surgery primary site, surgery metastasis site, survival time, survival status, and causes of death. For the indicator of race, the patients were categorized as white, black, American Indian/Alaska Native (AI), and Asian/Pacific Islander (API). For the grade and differentiation indicators, they were defined as well differentiated (code 1), moderately differentiated (code 2), poorly differentiated (code 3), or undifferentiated (code 4). The main observation indicators were overall survival (OS) and cancer-specific survival (CSS). OS was defined as the number of months from CRC diagnosis to either death from any cause or the end of follow-up. We defined CSS as the time from CRC diagnosis to death from CRC. The data released by the SEER database included the informed consent of the patients and were available by download for free, so medical ethical review and informed consent of the patients were not required.

### 2.2 Statistical Analysis

Age was changed from a continuous variable to a categorical variable using X-tile software and divided into 4 groups: 18–44, 45–64, 65–84, and >85 years. Tumors were also classified into 2 size-related groups by X-tile software: 0–5 and >5 cm. CRC patients with distant metastasis used to construct nomograms were randomly divided into the training group and validation group at a ratio of 7:3 through the “caTools” package in R software. The patients’ demographic and tumor characteristics were summarized with descriptive statistics. Comparisons of categorical variables were calculated using the chi-square test between patients with and without metastases. OS estimates were performed using the Kaplan–Meier method with the log-rank test. Also, the median survival time of patients in all different metastasis subtypes was estimated, especially for those with cranial and extracranial metastases. The multivariable Cox regression was obtained to identify covariates associated with increased all-cause mortality (ACM) using the significant factors (*p* < 0.05) in the univariate Cox regression model. Survival time was calculated from the date of diagnosis to the death or the last follow-up. Afterward, CRC-specific mortality (CSM) and CSS were assessed by using Fine and Gray’s competing risk regression. Cancer-specific cause of death was obtained from the SEER cause of death data. Furthermore, the multivariable Fine and Gray’s competing risk regression based on proportional subdistribution hazard models was performed and included the same covariates as used in the Cox regression analysis. In addition, the nomograms of OS and CSS prediction models were constructed based on the Cox proportional risk model and competitive risk model, respectively, and the discrimination of nomograms was evaluated by concordance index (C-index), and the consistency of models was evaluated by the calibration curve.

The relevant data were obtained from the SEER database using SEER*Stat 8.3.5 software (Surveillance Research Program, National Cancer Institute). All statistical analyses were performed using R 4.1.0 software (www.r-project.org). In all regressions, adjusted hazard ratios (HRs) and their 95% confidence intervals (95% CIs) of different covariates were reported. Statistical significance was set at two-sided *p* < 0.05.

### 2.3 Bioinformatics Analysis

In order to further explore the relevant mechanism of CRC distant metastasis from the genotype, we downloaded the gene expression profile datasets, GSE41258, from the GEO database (https://www.ncbi.nlm.nih.gov/geo/), submitted by Michal Sheffer in October 2012. GSE41258 had a total of 390 samples from CRC patients, including primary colon adenocarcinomas, adenomas, metastasis, and corresponding normal mucosae. GSE41258 dataset was grouped by the GEO2R online tool (https://www.ncbi.nlm.nih.gov/geo/geo2r/), and DEGs were analyzed. The upregulated and downregulated genes were obtained under the conditions of *p* < 0.05 and |logFC| > 1.5. The latest Kyoto Encyclopedia of Genes and Genomes (KEGG) Pathway gene annotation was obtained by using KEGG rest API (https://www.kegg.jp/kegg/rest/keggapi.html), and the Gene Ontology (GO) annotation in R software package org.hs.eg.db (version 3.1.0) and clusterProfiler (version 3.14.3) were used for enrichment analysis. The minimum gene set was set to 5, and *p* < 0.05 was considered statistically significant. The screened DEGs were imported into the String online database (https://string-db.org/) to construct the protein–protein interaction (PPI) network. Cytoscape 3.9.0 was used for further visualization. The cytoHubba plug-in was used to identify hub genes. The Molecular Complex Detection (MCODE) plug-in was used to screen modules of the PPI network in Cytoscape with a degree cutoff = 2, node score cutoff = 0.2, k-core = 2, and max depth = 100.

## 3 Results

### 3.1 Distant Metastasis Pattern and Prognostic Risk Factors

#### 3.1.1 Incidence

The study group consisted of 57,835 patients, including 30,199 men (52.2%) and 27,636 women (47.8%). A total of 10,012 CRC patients were diagnosed with distant metastasis (17.31%), and the incidence of CRC patients with distant metastasis in the right colon, left colon, and rectum was 7.69%, 5.38%, and 4.24%, respectively. Insured CRC patients were found in 55,682 (96.28%) cases, compared with 2,153 uninsured CRC patient cases (3.72%). The incidence rate of CRC patients with adenocarcinoma was 10.62 times greater than the others in the current cohort. Among the 57,835 patients with CRC grade analyzed for incidence, 7.32%, 73.72%, 16.02%, and 2.94% were well differentiated, moderately differentiated, poorly differentiated, and undifferentiated, respectively. As for serum CEA levels in CRC patients, 55.75%, 43.81%, and 0.53% were normal, elevated, and borderline, respectively.

CRC patients with distant metastasis had significant differences (all *p* < 0.01) in the age, race, insurance status, histology type, primary tumor sites, grade, tumor size, T stage, N stage, and serum CEA level as compared with patients without metastasis, but there was no statistical difference in gender (*p* = 0.13) and married status (*p* = 0.09). The specific clinical characteristics of CRC patients with or without metastases are represented in **Table 1**.

**Table 1:** Clinical characteristics of patients with or without metastasis.

Variable	Without metastasis	With metastasis	*p*-Value
	Number (%)	Number (%)	
Total	47,823 (82.69)	10,012 (17.31)	
**Age at diagnosis, year**			<0.01
18–45	3,703 (7.7)	1,137 (11.4)	
45–65	20,526 (42.9)	5,017 (50.1)	
65–85	20,207 (42.3)	3,444 (34.4)	
>85	3,387 (7.1)	410 (4.1)	
**Gender**			0.13
Male	24,902 (52.1)	5,297 (52.9)	
Female	22,921 (47.9)	4,715 (47.1)	
**Race**			<0.01
White	37,442 (78.3)	7,592 (75.8)	
Black	5,360 (11.2)	1,447 (14.5)	
AI	361 (0.8)	76 (0.8)	
API	4,660 (9.7)	897 (9.0)	
**Married status**			0.09
Unmarried	20,762 (43.4)	4,440 (44.3)	
Married	27,061 (56.6)	5,572 (55.7)	
**Insurance**			<0.01
Uninsured	1,637 (3.4)	516 (5.2)	
Insured	46,186 (96.6)	9,496 (94.8)	
**Primary tumor sites**			<0.01
Right colon	21,409 (44.8)	4,445 (44.4)	
Left colon	12,982 (27.1)	3,113 (31.1)	
Rectum	13,432 (28.1)	2,454 (24.5)	
**Histology**			<0.01
Adenocarcinoma	43,955 (91.9)	8,904 (88.9)	
Others	3,868 (8.1)	1,108 (11.1)	
**Grade**			<0.01
Well	3,738 (7.8)	497 (5.0)	
Moderately	35,961 (75.2)	6,674 (66.7)	
Poorly	6,913 (14.5)	2,352 (23.5)	
Undifferentiated	1,211 (2.5)	489 (4.9)	
**Tumor size, cm**			<0.01
0–5	31,430 (65.7)	5,155 (51.5)	
>5	16,393 (34.3)	4,857 (48.5)	
**T stage**			<0.01
T1	5,054 (10.6)	611 (6.1)	
T2	7,822 (16.4)	273 (2.7)	
T3	28,076 (58.7)	5,096 (50.9)	
T4	6,871 (14.4)	4,032 (40.3)	
**N stage**			<0.01
N0	27,792 (58.1)	2,164 (21.6)	
N1	13,683 (28.6)	3,850 (38.5)	
N2	6,348 (13.3)	3,998 (39.9)	
**CEA**			<0.01
Normal	30,105 (63.0)	2,137 (21.3)	
Elevated	17,445 (36.5)	7,893 (78.3)	
Borderline	273 (0.6)	36 (0.4)	

AI, American Indian/Alaska Native; API, Asian/Pacific Islander; CEA, carcinoembryonic antigen.

#### 3.1.2 Survival Outcomes

**Figure 1:**
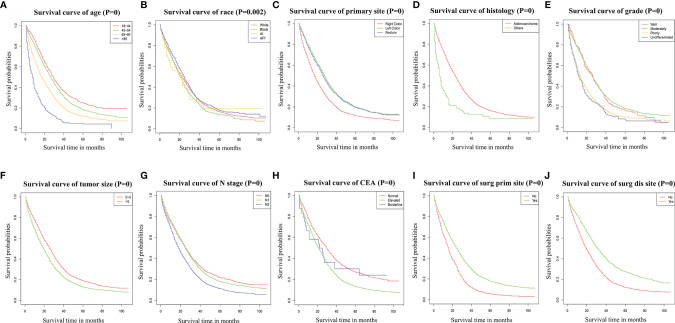
Survival curve of age **(A)**, race **(B)**, primary site **(C)**, histology **(D)**, grade **(E)**, tumor size **(F)**, N stage **(G)**, CEA **(H)**, Surg Prim Site **(I)**, and Surg Dis Site **(J)** of CRC patients with distant metastasis. *p* = 0: *p* < 0.0001. Surg Prim Site, primary site surgery; Surg Dis Site, distant metastasis site surgery; CEA, carcinoembryonic antigen; CRC, colorectal cancer.

**Figure 2:**
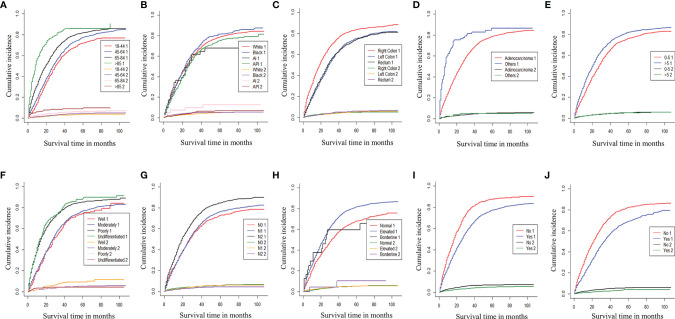
Cumulative incidence curve of age **(A)**, race **(B)**, primary site **(C)**, histology **(D)**, tumor size **(E)**, grade **(F)**, N stage **(G)**, CEA **(H)**, Surg Prim Site **(I)**, and Surg Dis Site **(J)** of CRC patients with distant metastases (1: cancer-specific death; 2: competitive death). Surg Prim Site, primary site surgery; Surg Dis Site, distant metastasis site surgery; CRC, colorectal cancer.

According to the Cox univariate analysis, age, race, primary site, history, grade, tumor size, N stage, CEA, primary site surgery, and distant metastasis site surgery were risk factors affecting OS in CRC patients with distant metastasis (*p* < 0.01). The survival curves of age (*p* < 0.001, **Figure 1A**), race (*p* = 0.002, **Figure 1B**), primary site (*p* < 0.001, **Figure 1C**), histology (*p* < 0.001, **Figure 1D**), grade (*p* < 0.001, **Figure 1E**), tumor size (*p* < 0.001, **Figure 1F**), N stage (*p* < 0.001, **Figure 1G**), CEA (*p* < 0.001, **Figure 1H**), primary site surgery (*p* < 0.001, **Figure 1I**), and distant metastasis site surgery (*p* < 0.001, **Figure 1J**) were drawn based on the Kaplan–Meier and log-rank tests. The variables with statistically significant differences in univariate analysis were further included in the multivariate analysis. The results showed that age ≥ 65 years (*p* < 0.001), black race (*p* < 0.001), primary tumor site in the right colon (*p* < 0.001), histology (*p* < 0.001), grade poorly (*p* < 0.001), tumor size >5 cm (*p* < 0.001), N1 (*p* = 0.002) or N2 stage (*p* < 0.001), CEA elevated (*p* < 0.001), no surgery at the primary site (*p* < 0.001), and no surgery at the distant metastasis site (*p* < 0.001) were independent risk factors for OS in CRC patients with distant metastasis (**Table 2**). After screening based on the Fine–Gray competitive risk model and multivariate analysis, the results showed that the independent risk factors related to patient CSS were age ≥ 65 years (*p* < 0.001), black race (*p* = 0.032), primary tumor site in the right colon (*p* < 0.001), history (*p* = 0.021), grade poorly (*p* < 0.001), tumor size >5 cm (*p* < 0.001), N1 (*p* = 0.019) or N2 stage (*p* < 0.001), CEA elevated (*p* < 0.001), no surgery at the primary site (*p* < 0.001), and no surgery at the distant metastasis site (*p* < 0.001) (**Table 2**). The cumulative incidence curves of age (**Figure 2A**), race (**Figure 2B**), primary site (**Figure 2C**), histology (**Figure 2D**), tumor size (**Figure 2E**), grade (**Figure 2F**), N stage (**Figure 2G**), CEA (**Figure 2H**), primary site surgery (**Figure 2I**), and distant metastasis site surgery (**Figure 2J**) were drawn.

**Table 2:** Univariate and multivariate analyses of OS and CSS in CRC patients with distant metastasis.

Variables	Univariate analysis of OS		Multivariate analysis of OS		Multivariate analysis of CSS	
	HR (95% CI)	*p*-Value	HR (95% CI)	*p*-Value	HR (95% CI)	*p*-Value
Age, years						
18–44	Reference		Reference		Reference	
45–64	1.19 (1.08–1.32)	<0.001*	1.14 (1.02–1.26)	0.016*	1.11 (1.00–1.23)	0.058
65–84	1.69 (1.52–1.88)	<0.001*	1.52 (1.37–1.70)	<0.001*	1.35 (1.21–1.51)	<0.001*
>85	3.33 (2.86–3.89)	<0.001*	3.08 (2.63–3.61)	<0.001*	2.14 (1.75–2.62)	<0.001*
Gender						
Female	Reference		–	–		
Male	1.01 (0.95–1.07)	0.814	–	–		
Race						
White	Reference		Reference		Reference	
Black	1.10 (1.02–1.19)	0.012*	1.15 (1.06–1.24)	<0.001*	1.09 (1.01–1.18)	0.032*
AI	1.04 (0.74–1.48)	0.810	1.24 (0.87–1.76)	0.227	0.94 (0.63–1.43)	0.780
API	0.93 (0.84–1.03)	0.142	0.98 (0.89–1.09)	0.745	0.93 (0.83–1.05)	0.240
Primary site						
Right colon	Reference		Reference		Reference	
Left colon	0.67 (0.63–0.71)	<0.001*	0.76 (0.71–0.81)	<0.001*	0.77 (0.72–0.82)	<0.001*
Rectum	0.69 (0.64–0.74)	<0.001*	0.62 (0.56–0.68)	<0.001*	0.63 (0.58–0.69)	0.000*
Histology						
Adenocarcinoma	Reference		Reference		Reference	
Others	2.02 (1.61–2.54)	<0.001*	1.50 (1.18–1.88)	<0.001*	1.47 (1.06–2.03)	0.021*
Grade						
Well	Reference		Reference		Reference	
Moderately	0.96 (0.83–1.10)	0.552	1.02 (0.89–1.18)	0.740	1.12 (0.97–1.30)	0.110
Poorly	1.54 (1.33–1.79)	<0.001*	1.52 (1.31–1.77)	<0.001*	1.62 (1.39–1.90)	<0.001*
Undifferentiated	1.72 (1.43–2.08)	<0.001*	1.66 (1.37–2.00)	<0.001*	1.68 (1.37–2.07)	<0.001*
Tumor size, cm						
0–5	Reference		Reference		Reference	
>5	1.28 (1.21–1.36)	<0.001*	1.26 (1.19–1.33)	<0.001*	1.23 (1.16–1.31)	<0.001*
N stage						
N0	Reference		Reference		Reference	
N1	1.05 (0.97–1.14)	0.196	1.14 (1.05–1.23)	0.002*	1.11 (1.02–1.20)	0.019*
N2	1.42 (1.31–1.53)	<0.001*	1.54 (1.42–1.67)	<0.001*	1.51 (1.39–1.65)	0.000*
CEA						
Normal	Reference		Reference		Reference	
Elevated	1.49 (1.38–1.60)	<0.001*	1.50 (1.39–1.62)	<0.001*	1.44 (1.33–1.56)	0.000*
Borderline	1.18 (0.73–1.91)	0.496	1.14 (0.70–1.84)	0.602	0.98 (0.54–1.77)	0.950
Surg Prim Site						
No	Reference		Reference		Reference	
Yes	0.60 (0.56–0.65)	<0.001*	0.47 (0.43–0.52)	<0.001*	0.53 (0.49–0.59)	0.000*
Surg Dis Site						
No	Reference		Reference		Reference	
Yes	0.61 (0.57–0.66)	<0.001*	0.71 (0.66–0.76)	<0.001*	0.76 (0.71–0.82)	<0.001*

Surg Prim Site, primary site surgery; Surg Dis Site, distant metastasis site surgery; HR, hazard ratio; OS, overall survival; CSS, cancer-specific survival; CRC, colorectal cancer; HR, hazard ratio; AI, American Indian/Alaska Native; API, Asian/Pacific Islander.

^*^p < 0.05.

In addition, the Kaplan–Meier analysis showed that OS in subjects with liver metastases (*p* < 0.01, **Supplementary Figure 2A**), lung metastases (*p* < 0.01, **Supplementary Figure 2B**), bone metastases (*p* < 0.01, **Supplementary Figure 2C**), and brain metastases (*p* < 0.01, **Supplementary Figure 2D**) was shorter than that of their counterparts. The number of extracranial metastasis organs was also associated with lower decreased survival incidence, as shown in **Supplementary Figure 3**. Meanwhile, **Table 3** shows the median survival time of patients by subtype stratified by system disease severity. In general, patients with more extracranial disease at the time of diagnosis have lower survival rates. We also found that brain metastases at newly diagnosed patients were associated with shorter survival times compared with patients only with metastatic disease rather than brain metastases (**Table 3**).

**Table 3:** Median survival of CRC patients by extent of systemic metastatic disease.

Subtype	Type of metastasis	Extracranial systemic disease only		Extracranial systemic disease and brain metastases	
		Number	Median survival months	Number	Median survival months
Right colon	No	22,632	NR (NR–NR)	21	5 (3–NR)
	Liver	2,468	17 (16–18)	9	2 (1–NR)
	Lung	180	25 (19–30)	3	10.0 (10–NR)
	Bone	26	10 (5–NR)	1	22 (NR–NR)
	2 of 3	483	11 (9–13)	11	14 (7–NR)
	all 3	52	6 (3–12)	2	7 (3–NR)
Left colon	No	13,516	NR (NR–NR)	9	9 (2–NR)
	Liver	1,975	28 (27–30)	2	4 (1–NR)
	Lung	167	27 (24–39)	4	1.5 (1–NR)
	Bone	28	4.5 (3–NR)	1	1 (NR–NR)
	2 of 3	388	18 (16–21)	7	15 (3–NR)
	All 3	39	8 (5–22)	2	2 (1–NR)
Rectum	No	13,813	NR (NR–NR)	10	11 (5–NR)
	Liver	1,328	29 (27–31)	0	NR (NR–NR)
	Lung	249	30 (25–35)	4	18 (7–NR)
	Bone	30	12 (5–NR)	1	NR (NR–NR)
	2 of 3	420	16 (15–18)	7	8 (7–NR)
	All 3	41	7 (5–10)	2	13 (7–NR)

CRC, colorectal cancer; NR, not reached.

#### 3.1.3 Analyses of Mortalities of Patients With Metastases

All 10,012 CRC patients with metastases were included to analyze mortalities. On the multivariable Cox regression analysis for ACM among patients with distant metastasis in newly diagnosed CRC, older age (>85 years old: HR 3.62, 95% CI 3.15–4.16, *p* < 0.01) was related to a higher ACM. In addition, larger tumor size (>5 cm: HR 1.16, 95% CI 1.10–1.22, *p* < 0.01), N2 stage (HR 1.22, 95% CI 1.14–1.32, *p* < 0.01), elevated CEA (HR 1.51, 95% CI 1.41–1.62, *p* < 0.01), and poorly differentiated grade (HR 1.85, 95% CI 1.57–2.18, *p* < 0.01) were also significantly associated with an increased ACM (**Table 4**). As for distant metastases, more metastatic sites (HR 3.56, 95% CI 2.90–4.38, *p* < 0.01) and brain metastases were related to a poorer prognosis. It should be noted that married status, which was significantly associated with a decreased ACM, might be a potential protective factor of patients’ prognosis.

**Table 4:** All-cause mortality and CRC-specific mortality among patients with metastases.

Variable	All-cause mortality		Cancer-specific mortality	
	Hazard ratio (95% CI)	*p*-Value	Hazard ratio (95% CI)	*p*-Value
**Age at diagnosis, year**				
18–45	Reference		Reference	
45–65	1.21 (1.11–1.33)	<0.01	1.18 (1.08–1.28)	<0.01
65–85	1.83 (1.66–2.01)	<0.01	1.62 (1.48–1.78)	<0.01
>85	3.62 (3.15–4.16)	<0.01	2.57 (2.16–3.06)	<0.01
**Married status**				
Unmarried	Reference		Reference	
Married	0.77 (0.73–0.81)	<0.01	0.80 (0.75–0.84)	<0.01
**Primary tumor sites**				
Right colon	Reference		Reference	
Left colon	0.75 (0.70–0.79)	<0.01	0.75 (0.70–0.80)	<0.01
Rectum	0.78 (0.73–0.84)	<0.01	0.79 (0.74–0.85)	<0.01
**Histology**				
Adenocarcinoma	Reference		Reference	
Others	1.07 (0.99–1.17)	0.98	1.10 (1.00–1.20)	<0.05
**Grade**				
Well	Reference		Reference	
Moderately	1.11 (0.97–1.26)	0.13	0.91 (0.97–1.25)	0.15
Poorly	1.76 (1.54–2.01)	<0.01	1.75 (1.53–2.00)	<0.01
Undifferentiated	1.85 (1.57–2.18)	<0.01	1.75 (1.47–2.08)	<0.01
**Tumor size, cm**				
0–5	Reference		Reference	
>5	1.16 (1.10–1.22)	<0.01	1.15 (1.09–1.21)	<0.01
**N stage**				
N0	Reference		Reference	
N1	1.03 (0.96–1.11)	0.44	1.03 (0.96–1.11)	0.43
N2	1.22 (1.14–1.32)	<0.01	1.24 (1.15–1.33)	<0.01
**CEA**				
Normal	Reference		Reference	
Elevated	1.51 (1.41–1.62)	<0.01	1.44 (1.34–1.54)	<0.01
Borderline	1.60 (1.07–2.40)	0.02	1.56 (1.03–2.34)	0.03
**Extracranial metastatic sites to bone, lung, and liver, no.**				
0 site	Reference		Reference	
1 site	1.17 (1.10–1.26)	<0.01	1.19 (1.11–1.29)	<0.01
2 sites	1.91 (1.74–2.09)	<0.01	1.92 (1.74–2.12)	<0.01
3 sites	3.56 (2.90–4.38)	<0.01	3.24 (2.54–4.12)	<0.01
**Brain metastasis**				
No	Reference		Reference	
Yes	2.01 (1.60–2.53)	<0.01	1.70 (1.24–2.34)	<0.01

CRC, colorectal cancer; CEA, carcinoembryonic antigen.

Similarly, when CSM was performed using the multivariable competing-risk analysis, the results were the same. However, mucinous adenocarcinoma and SRCC were related to an increased CSM than adenocarcinoma (HR 1.10, 95% CI 1.00–1.20, *p* < 0.05). All results of ACM and CSM analyses are presented in **Table 4**.

### 3.2 Construction and Verification of Nomogram Prediction Model

#### 3.2.1 Construction of Nomogram Prediction Model

In this study, 10,012 CRC patients with distant metastasis from 2010 to 2016 were randomly divided into the training group (n = 7,008) and validation group (n = 3,004) according to the ratio of 7:3. There was no significant difference between the two groups in diagnosis year, age, gender, race, primary site, pathological type, number of detected lymph nodes, metastasis, tumor size, histological grade, T stage, N stage, primary site operation, and distant metastasis site operation (*p* > 0.05), so the random grouping of the training group and the validation group was comparable.

**Figure 3:**
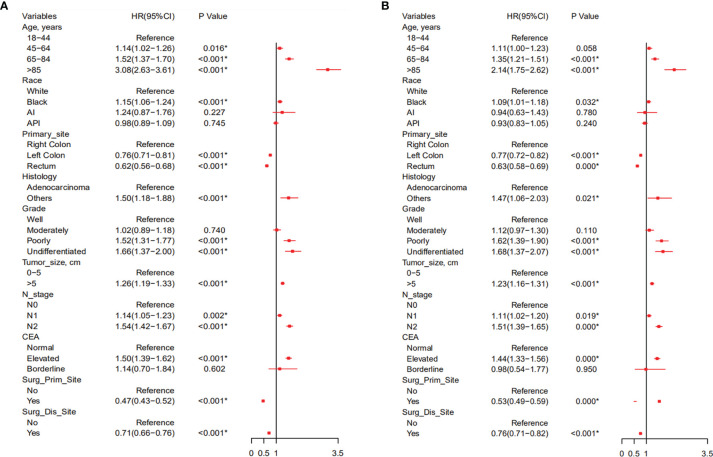
Forest plot of all variables with hazard ratios in CRC patients with distant metastasis with OS **(A)** and CSS **(B)**. CRC, colorectal cancer; OS, overall survival; CSS, cancer-specific survival.

**Figure 4:**
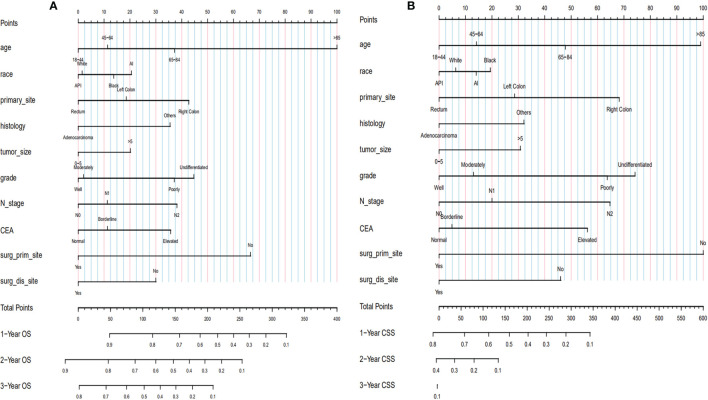
Establishment of nomograms regarding both OS **(A)** and CSS **(B)**. OS, overall survival; CSS, cancer-specific survival.

Based on the selected independent risk factors affecting patients’ OS (**Figure 3A**) and CSS (**Figure 3B**), we constructed nomogram models to predict patients’ OS (**Figure 4A**) and CSS (**Figure 4B**). In the prediction models, the risk factor scores (**Table 5**) were added together to obtain a total score, and the value corresponding to the total score could be used to predict a patient’s 1-, 2-, and 3-year OS and CSS.

**Table 5:** Scores of prognostic factors in the OS and CSS nomograms.

Variables	OS nomogram	CSS nomogram	Variables	OS nomogram	CSS nomogram
Age, years			Grade		
18–44	0	0	Well	0	0
45–64	11	14	Moderately	2	13
65–84	37	48	Poorly	37	64
>85	100	99	Undifferentiated	45	74
Race			N stage		
White	2	6	N0	0	0
Black	14	19	N1	11	20
AI	21	14	N2	38	65
API	0	0	CEA		
Primary site			Normal	0	0
Right colon	43	68	Elevated	36	56
Left colon	19	29	Borderline	11	5
Rectum	0	0	Surg Prim Site		
Histology			No	67	100
Adenocarcinoma	0	0	Yes	0	0
Others	35	32	Surg Dis Site		
Tumor size, cm			No	30	46
0-5	0	0	Yes	0	0
>5	20	31			

Surg Prim Site, primary site surgery; Surg Dis Site, distant metastasis site surgery; OS, overall survival; CSS, cancer-specific survival; AI, American Indian/Alaska Native; API, Asian/Pacific Islander.

#### 3.2.2 Verification of Nomogram Prediction Model

**Figure 5:**
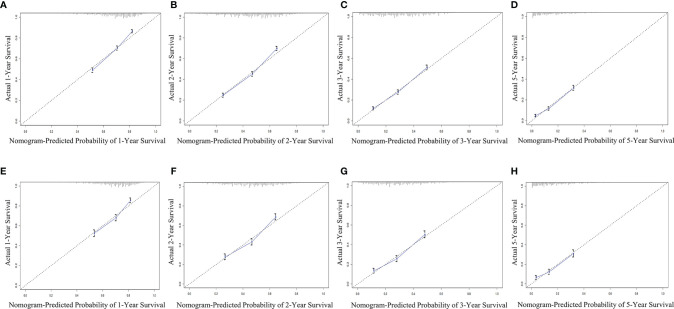
Evaluation of calibration plots based on OS of the training group **(A–D)** and the validation group **(E–H)** in 1, 2, 3, and 5 years. The slanted gray line represents an ideal match between the actual survival (y-axis) and nomogram-predicted survival (x-axis). The perpendicular line means 95% CIs. Closer distances from the points to the dashed line indicate higher prediction accuracy. OS, overall survival.

The bootstrap method was used to repeatedly sample 1,000 times to verify the modeling effect of the nomogram. The C-index of the OS nomogram prediction model in the training and validation groups was 0.67 (95% CI 0.662–0.678) and 0.658 (95% CI 0.646–0.670), respectively. In addition, the prediction curve and the ideal curve in the calibration diagrams of the training group (**Figures 5A–D**) and validation group (**Figures 5E–H**) fitted well, indicating that the model of OS had good accuracy.

**Figure 6:**
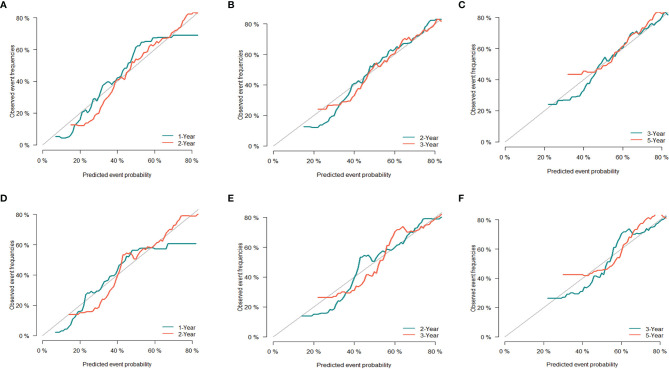
Evaluation of calibration plots based on CSS of the training group **(A–C)** and the validation group **(D–F)** in 1, 2, 3, and 5 years. The 45° gray line represents the ideal curve, and the colored line represents the nomogram. CSS, cancer-specific survival.

The C-index of the CSS nomogram prediction model in the training and validation groups was 0.692 (95% CI 0.682–0.702) and 0.646 (95% CI 0.622–0.670), respectively. In addition, the calibration curves of the training group (**Figures 6A–C**) and validation group (**Figures 6D–F**) also showed a good fit between the predicted curve and the ideal curve, indicating that the model of CSS had a high degree of calibration.

### 3.3 Bioinformatics Analysis

#### 3.3.1 Differentially Expressed Gene Screening

**Figure 7:**
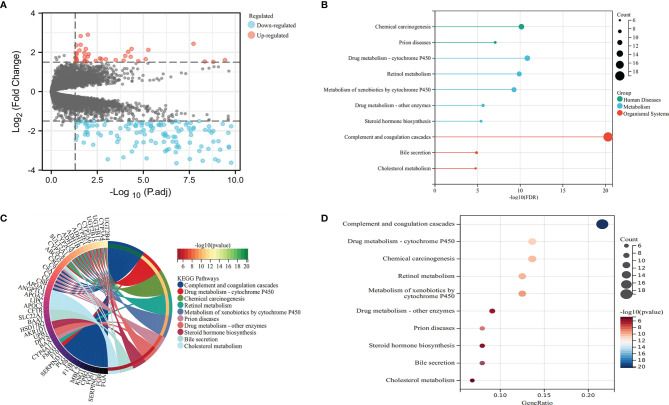
Volcano map **(A)** and KEGG enrichment plots **(B–D)** of DEG expression. KEGG, Kyoto Encyclopedia of Genes and Genomes; DEG, differentially expressed gene.

Five samples of primary colon tumor tissue of CRC patients with liver metastasis and 178 samples of primary colon tumor tissue of CRC patients without liver metastasis were selected from the GSE41258 datasets. Then 158 eligible DEGs were obtained by GEO2R online analysis, including 37 upregulated genes and 121 downregulated genes. The top 10 upregulated and downregulated DEGs with the most significant differential expression were obtained according to the size of |logFC| (**Table 6**). The DEG expression is visually displayed by a volcano map (**Figure 7A**).

**Table 6:** Top ten upregulated and downregulated DEGs.

Ranks	Upregulated		Downregulated	
	Gene symbol	Gene title	Gene symbol	Gene title
1	*CXCL11*	C-X-C motif chemokine ligand 11	*ALB*	Albumin
2	*CFTR*	Cystic fibrosis transmembrane conductance regulator	*APOC3*	Apolipoprotein C3
3	*ATP13A2*	ATPase 13A2	*LBP*	Lipopolysaccharide binding protein
4	*MMP1*	Matrix metallopeptidase 1	*BAAT*	Bile acid-CoA:amino acid *N*-acyltransferase
5	*NOX1*	NADPH oxidase 1	*ORM1*	Orosomucoid 1
6	*LRFN4*	Leucine-rich repeat and fibronectin type III domain containing 4	*FGB*	Fibrinogen beta chain
7	*BAX*	BCL2 associated X, apoptosis regulator	*FGG*	Fibrinogen gamma chain
8	*FKBP10*	FK506 binding protein 10	*APOA2*	Apolipoprotein A2
9	*CENPM*	Centromere protein M	*ORM1*	Orosomucoid 1
10	*NPTX2*	Neuronal pentraxin 2	*HPD*	4-Hydroxyphenylpyruvate dioxygenase

#### 3.3.2 Gene Ontology and Kyoto Encyclopedia of Genes and Genomes Analyses

The GO analysis results of DEGs were as follows. 1) Biological processes (BP): DEGs were mainly concentrated in protein activation cascade, acute inflammatory response, cytolysis, platelet degranulation, steroid metabolic process, blood coagulation, fibrin clot formation, complement activation, fibrinolysis, etc. 2) Cellular components (CC): DEGs were mainly concentrated in blood microparticle, extracellular region, extracellular space, extracellular exosome, endoplasmic reticulum lumen, extracellular vesicle, etc. 3) Molecular functions (MF): DEGs were mainly concentrated in enzyme inhibitor activity, heparin binding, endopeptidase, inhibitor activity, signaling receptor binding, high-density lipoprotein particle receptor binding, oxidoreductase activity, etc. (**Supplementary Table 1**). KEGG pathway analysis showed that DEGs were mainly concentrated in complement and coagulation cascades, drug metabolism—cytochrome P450, chemical carcinogenesis, retinol metabolism, metabolism of xenobiotics by cytochrome P450, prion diseases, drug metabolism—other enzymes, steroid hormone biosynthesis, bile secretion, cholesterol metabolism, etc. (**Figures 7B–D**).

#### 3.3.3 Protein–Protein Interaction Analysis and Hub Genes

**Figure 8:**
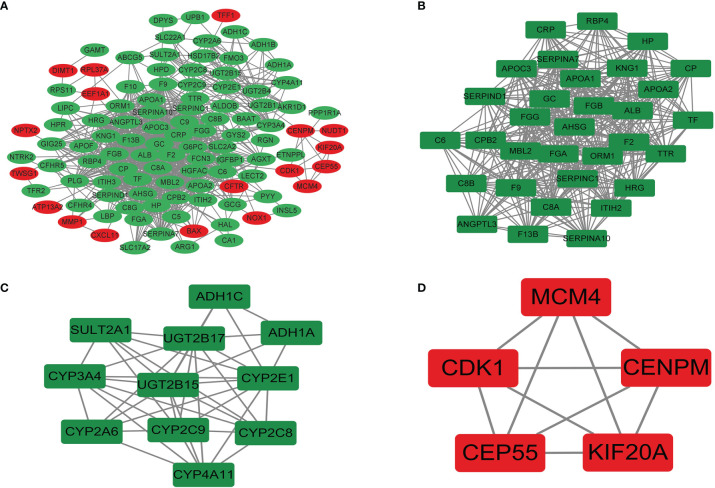
The PPI network **(A)** and significant interaction modules **(B–D)** of DEGs. Red, upregulated; green, downregulated. PPI, protein–protein interaction; DEGs, differentially expressed genes.

The PPI network was preliminarily obtained by using the String online website. The results were imported into Cytoscape 3.9.0 for further analysis to obtain 108 protein nodes and 863 edges, and the PPI network was drawn (**Figure 8A**). Significant interaction modules 1, 2, and 3 (**Figures 8B–D**) were obtained by using the MCODE plug-in, each containing 32, 11, and 5 nodes and 388, 40, and 10 edges, with MCODE score of 25.032, 8, and 5, respectively. Ten hub genes were screened by 8 different algorithms of cytohubba plug-in (**Table 7**). According to the Degree algorithm, the hub genes were albumin (ALB), fibrinogen alpha chain (FGA), alpha 2-HS glycoprotein (AHSG), coagulation factor II (F2), apolipoprotein C3 (APOC3), serpin family C member 1 (SERPINC1), fibrinogen gamma chain (FGG), fibrinogen beta chain (FGB), apolipoprotein A1 (APOA1), and vitamin D binding protein (GC). KEGG analysis of hub genes showed that they were mainly concentrated in liver function and coagulation function.

**Table 7:** Top 10 hub genes.

Ranks	Genes by MCC	Genes by DMNC	Genes by MNC	Genes by Degree	Genes by EPC	Genes by BottleNeck	Genes by EcCentricity	Genes by Closeness
1	*ITIH2*	*ITIH2*	*ALB*	*ALB*	*ALB*	*ALB*	*ALB*	*ALB*
2	*KNG1*	*SERPIND1*	*FGA*	*FGA*	*FGA*	*TTR*	*PLG*	*FGA*
3	*GC*	*HGFAC*	*AHSG*	*AHSG*	*AHSG*	*GC*	*ITIH2*	*AHSG*
4	*HRG*	*KNG1*	*F2*	*F2*	*SERPINC1*	*ITIH2*	*FGA*	*F2*
5	*SERPINC1*	*SERPINA7*	*APOC3*	*APOC3*	*F2*	*FGA*	*AHSG*	*APOC3*
6	*APOA2*	*SERPINA10*	*SERPINC1*	*SERPINC1*	*ORM1*	*F2*	*F2*	*TTR*
7	*FGB*	*GIG25*	*FGG*	*FGG*	*FGG*	*MBL2*	*APOC3*	*SERPINC1*
8	*FGG*	*GC*	*FGB*	*FGB*	*APOC3*	*CYP2C8*	*TTR*	*FGG*
9	*ORM1*	*CP*	*APOA1*	*APOA1*	*FGB*	*EEF1A1* ^*^	*SERPINC1*	*FGB*
10	*TF*	*HRG*	*GC*	*GC*	*HRG*	*UGT2B17*	*FGG*	*APOA1*

*denotes upregulatory gene, and others are downregulatory genes.

## 4 Discussion

CRC was the third most common cancer among the population in the United States. With the development of diagnostic techniques, diagnostic accuracy for metastatic CRC will be greatly improved, and more metastatic diseases are found than ever before. Furthermore, it was suggested by Bailey et al. that although the incidence of CRC has been decreasing in older persons, the incidence was increasing significantly in young adults (8). So it is very important to identify the related factors and screen effects for distant metastasis development among high-risk CRC patients. In this study, the clinical and pathological features of CRC patients were described at initial diagnosis with or without distant metastases from the SEER database. Then we also characterize the risk factors and subsequent survival of identified distant metastases among patients with newly diagnosed CRC, which may have significant implications for clinical decision-making.

A nomogram is a visual statistical graph used to predict the prognosis of various diseases. It can score each independent risk factor based on the results of multivariate analysis, and the sum of the points of each factor corresponds to the incidence of the endpoint event, so as to predict the probability of the patient’s endpoint event (9). In the present study, a nomogram model was constructed to predict OS and CSS in CRC patients with distant metastasis, and the model’s C-indices were approximately 0.67 and 0.69, respectively. Considering that the distant metastatic tumor is affected by confounding variables, the predictive ability of the model constructed in this study could be considered relatively good. By including the patient’s personal clinical and pathological information, the model constructed herein could briefly and intuitively predict the patient’s OS and CSS.

Based on the results of our analysis, some relevant clinical factors including age, race, and insurance status might be related to synchronous distant metastasis in CRC as well as a few cancer-related covariates such as histology type, primary tumor sites, differentiated grade, tumor size, T stage, N stage, and serum CEA level. These results were partly consistent with a previous study (10). Hugen et al. (11) found that in histological subtypes, the proportion of MC and SRCC metastasis is higher, which was similar to our study. Also, we found that CRC patients without insurance were more likely to metastasize than insured patients. Insured patients represent people with social security, such as Medicare. One hypothesis is undertreatment and absence of psychosocial support as reasons for more distant metastases in uninsured patients. So we should give more care and social support to uninsured patients so that patients can improve their wellbeing, improve their mental health, and reduce the occurrence of metastasis. Therefore, for patients with CRC who have the above factors, it is important to be vigilant at the time of diagnosis. However, the present study showed that gender was not associated with CRC patients with distant metastasis development, which was not consistent with the previous study (12), and more studies are warranted to further verify the results.

Despite extensive early screening for CRC, approximately 25% of CRC patients have distant metastases at the time of diagnosis (13, 14). A quite low rate of brain metastases at the time of diagnosis has been reported in prior studies, only 0.2% in this dataset (15). Some reported incidence of bone metastases ranged from 0.96% to 11.1% in CRC patients (16, 17). In addition, it was suggested that the distant metastasis site was associated with the primary site. For colon cancer patients, there is a high incidence of abdominal metastases, whereas for rectal cancer patients, there are more extra-abdominal metastases such as in the lungs and brain (11). In our study, the liver was the most common metastatic site in the four sites (7,165/57,835, 12.39%), followed by the lungs (1,897/57,835, 3.28%), bone (342/57,835, 0.59%), and brain (96/57,835, 0.17%), the proportion of which was consistent with the finding (18).

The prognosis of metastatic CRC is poor. According to the results of analysis based on SEER, the 5-year relative survival rate is 13.5%, which is much lower than that of other high-metastasis cancers such as breast cancer. Our results, based on a large population analysis in the real world, identified a distant metastasis and poor prognosis in patients with a median OS of 30 months at most. The more metastatic sites in CRC patients, the worse their prognosis was. It should be noted that once the patients were diagnosed with brain metastasis, their median survival time is shorter than those with extracranial metastases. Compared with other organ metastases, lung metastases grow slower, and the OS rate is higher. So lung metastasis is the subtype with the best prognosis among metastasis types (19). However, patients with bone or brain metastases had the worst median survival and had little progress over time, which is similar to the prior studies (20, 21). Moreover, it was shown in our study that the prognosis of patients with rectal cancer and left colon cancer was better significantly than that of persons with right colon cancer. The reason might be that they have different embryonic sources, which affect biological habits, leading to significantly different epidemiology and clinical manifestations (22–25).

A series of prognostic factors for CRC patients with distant metastasis were found in the present research. In this study, patients with older age, unmarried status, elevated serum CEA, larger tumor size, N2 stage, poorly differentiated or undifferentiated grade, more metastatic sites, and right colon site might be related to higher cancer-specific mortality, which was similar to the results from a prior study (15). TNM staging of CRC is an important factor that significantly affects distant metastasis. The larger the tumor, the greater the tendency to have distant metastasis, and the shorter the OS rate, as a study reported (26). Tumor grading and staging have a reference value for clinically developing treatment plans and estimating prognosis, and especially tumor staging is more important. It is obvious that the higher the stage of the CRC, such as the N2 stage and more metastatic sites, the worse the prognosis (27). Simultaneously, poor differentiation of the CRC indicates an unfavorable prognosis. Hsu et al. (10) reported that CEA is elevated in approximately 40% of CRC, and it is still an independent factor influencing survival. Moreover, the prognosis of unmarried patients is worse than that of married patients, probably because spouses can provide social support and encourage patients to seek medical attention (28). Also, it has been previously reported that age and ethnicity were factors that influence the prognosis of patients with distant metastasis of CRC (29). Chan et al. (30) believed that the prognosis of the younger group was worse in the older age group, which was inconsistent with this study, and more studies are needed to further confirm these results.

The abovementioned clinical prognostic factors were analyzed from the macro level through the SEER database. In order to further and comprehensively explore the mechanism of CRC metastasis, related differential genes were studied from the micro level through the GEO database, because by studying the macro influencing factors, clinicians can more reasonably predict the prognosis risk from the external characteristics of patients. At the same time, the study of micro gene levels could enable clinicians to make accurate treatment plans according to the pathogenesis of patients. In this way, the combination of macro and micro levels could better contribute to the diagnosis and treatment of patients. Therefore, we compared CRC tissues with liver metastasis and CRC tissues without metastasis by mining the GEO database and obtaining 158 DEGs. The top 10 hub genes were screened by the PPI network according to the Degree algorithm, and it was found that the hub genes were mainly concentrated in liver function and coagulation function. A prospective cohort study supported that there is a correlation between liver function and CRC (31). ALB is synthesized by liver substantive cells, and lower circulating levels of ALB are associated with a higher risk of CRC (31). AHSG is primarily produced by the liver. It can modulate the etiology of diabetes and other metabolic diseases (32) and promote the invasion of tumor cells (33). ApoA1 and APOC3 belong to the lipoprotein family, and their biological functions are mainly involved in lipoprotein metabolism and cholesterol transport (34). Studies have shown that lipoproteins play a role in the occurrence and development of various malignant tumors (35). A study showed that serum APOA1 in CRC was significantly reduced (36). A low serum APOA1 expression level was associated with poor survival and advanced stage in CRC (37). All SERPINC1, FGA, FGG, FGB, and F2 genes regulate the expression of coagulation factors. SERPINC1 inhibits thrombin-induced tumor growth and angiogenesis, impairing proliferation and migration of cancer cells (36). High levels of phosphorylated FGA have been observed in CRC tissues (38). FGA protein consists of 2 subunits, each composed of Aα, Bβ, and γ3 polypeptides encoded by FGA, FGB, and FGG genes, respectively (39). Proteolytic cleaving of F2 generates activated serine protease thrombin. Overproduction of thrombin not only increases blood coagulation but also promotes the growth and metastasis of tumors. A study indicated that LGR5^+^ cell expansion is a hallmark of CRC tumorigenesis occurring during progression to adenoma, which may be related to the change of glandular structure (40). In our study, it was also found that LGR5 gene increased, but it was not obvious enough, which may be affected by some confounding factors such as gene interaction.

The most significant module was filtered from the PPI network, among which the majority of the corresponding genes were mostly associated with complement and coagulation cascades. It was demonstrated that cancer increases the risk of thrombosis by 4.1-fold (41) and results in the hyperactivation of coagulation and clotting abnormalities in cancer. The evidence has demonstrated that hypercoagulation and activation of complement cascades promote the pro-tumorigenic phenotype of immune cells and protect tumor cells from immune attack, ultimately favoring tumor development, progression, and metastasis formation (42). As an important component of tumor-promoting inflammation, activation of the complement system promotes cancer cell proliferation, dedifferentiation, and migration (43). Furthermore, specific experimental and clinical evidence suggests a reciprocal interaction between complement and coagulation. Complement may induce hyperactivation of the coagulation cascade by modifying cellular membranes, inducing platelet activation and aggregation, and stimulating the production of tissue factors in human neutrophils (42).

This study is a recent comprehensive analysis of distant metastasis patterns and prognostic prediction models of CRC patients. The population-based nature of the registry mirrors the real-world outcomes. Mortalities of all causes and cancer-specific causes were reported over a 5-year period. Also, the sample size is larger enough in a high degree of statistical power. Nevertheless, regardless of the strengths mentioned above, this study has several limitations as well. First, it is necessary to mention that the SEER database does not contain some important information that was relevant to the diagnostic method of tumor metastases and treatment. Thus, the specific treatment modalities on the survival of CRC patients with distant metastasis could not be captured in our analysis. Then, we only have information on synchronous metastasis to the liver, lung, bone, and brain, a relative minority compared to that of patients who will develop metachronous lesions, which may lead to an underestimation of other sites of metastasis. Finally, this study constructed nomograms based on retrospective analysis, and the level of research evidence was low, so our findings need further verification through prospective studies. These limitations have to be weighed against the strengths of the presented analysis.

## 5 Conclusion

In this study, based on big data mining, we described the distant metastasis pattern of CRC, screened the risk factors, constructed prognosis prediction models, and explored the hub gene affecting liver metastasis. The findings hopefully could help clinicians identify newly diagnosed CRC patients with distant metastasis and deliver appropriate treatment.

## Data Availability Statement

Publicly available datasets were analyzed in this study. These data were derived from the following resources available in the public domain: Surveillance, Epidemiology, and End Results (SEER) (http://seer.cancer.gov/) and Gene Expression Omnibus (GEO) database (https://www.ncbi.nlm.nih.gov/geo/).

## Author Contributions

Conception and design: CL, JY, and TW. Administrative support: WD. Provision of study materials or patients: CL, JY, and TW. Collection and assembly of data: CL, JY, and TW. Data analysis and interpretation: CL, JY, and TW. Manuscript writing: all authors. Final approval of manuscript: all authors.

## Funding

The National Natural Science Foundation of China (No. 82170549 and 81572426) funded this study.

## Conflict of Interest

The authors declare that the research was conducted in the absence of any commercial or financial relationships that could be construed as a potential conflict of interest.

## Publisher’s Note

All claims expressed in this article are solely those of the authors and do not necessarily represent those of their affiliated organizations, or those of the publisher, the editors and the reviewers. Any product that may be evaluated in this article, or claim that may be made by its manufacturer, is not guaranteed or endorsed by the publisher.

## Supplementary Material

Supplementary Figure 1 Screening process.

Supplementary Figure 2 Overall survival of patients with newly diagnosed colorectal cancer and: **(A)** liver metastases; **(B)** lung metastases; **(C)** bone metastases and **(D)** brain metastases.

Supplementary Figure 3 Overall survival of patients with newly diagnosed colorectal cancer and extracranial metastases, which was classified by the number of metastatic sites to the bone, lung, or liver.

Supplementary Table 1 GO and KEGG enrichment analysis of DEGs.
