# Supplementary figures and images for: Distant Metastasis Pattern and Prognostic Prediction Model of Colorectal Cancer Patients Based on Big Data Mining
Source: Front Oncol. 2022 Apr 22;12:878805. doi: 10.3389/fonc.2022.878805 (PMC9074728; doi:10.3389/fonc.2022.878805)

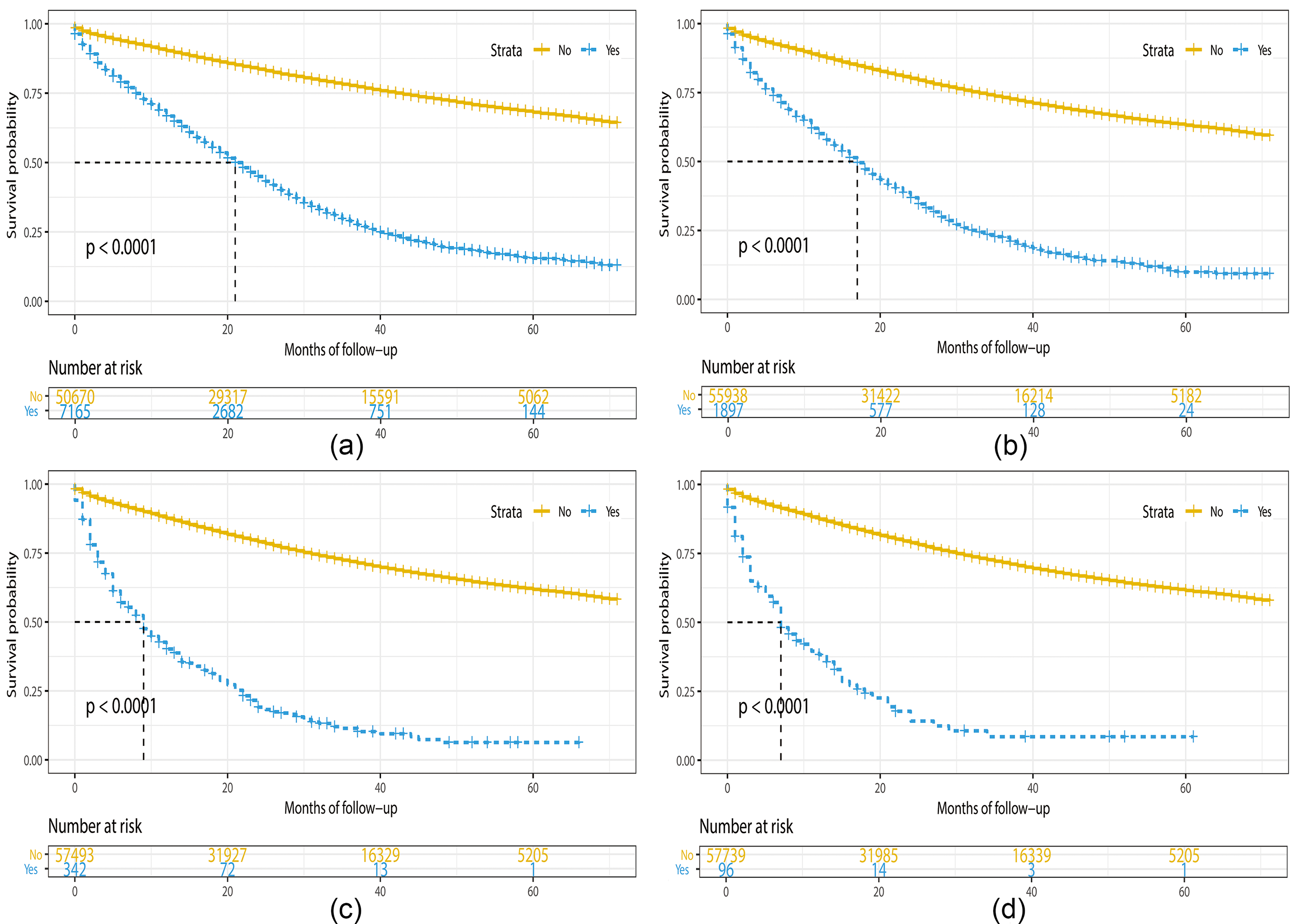

Supplement: Supplementary Figure 2 — Overall survival of patients with newly diagnosed colorectal cancer and: (A) liver metastases; (B) lung metastases; (C) bone metastases and (D) brain metastases. [file Image_2.tif]

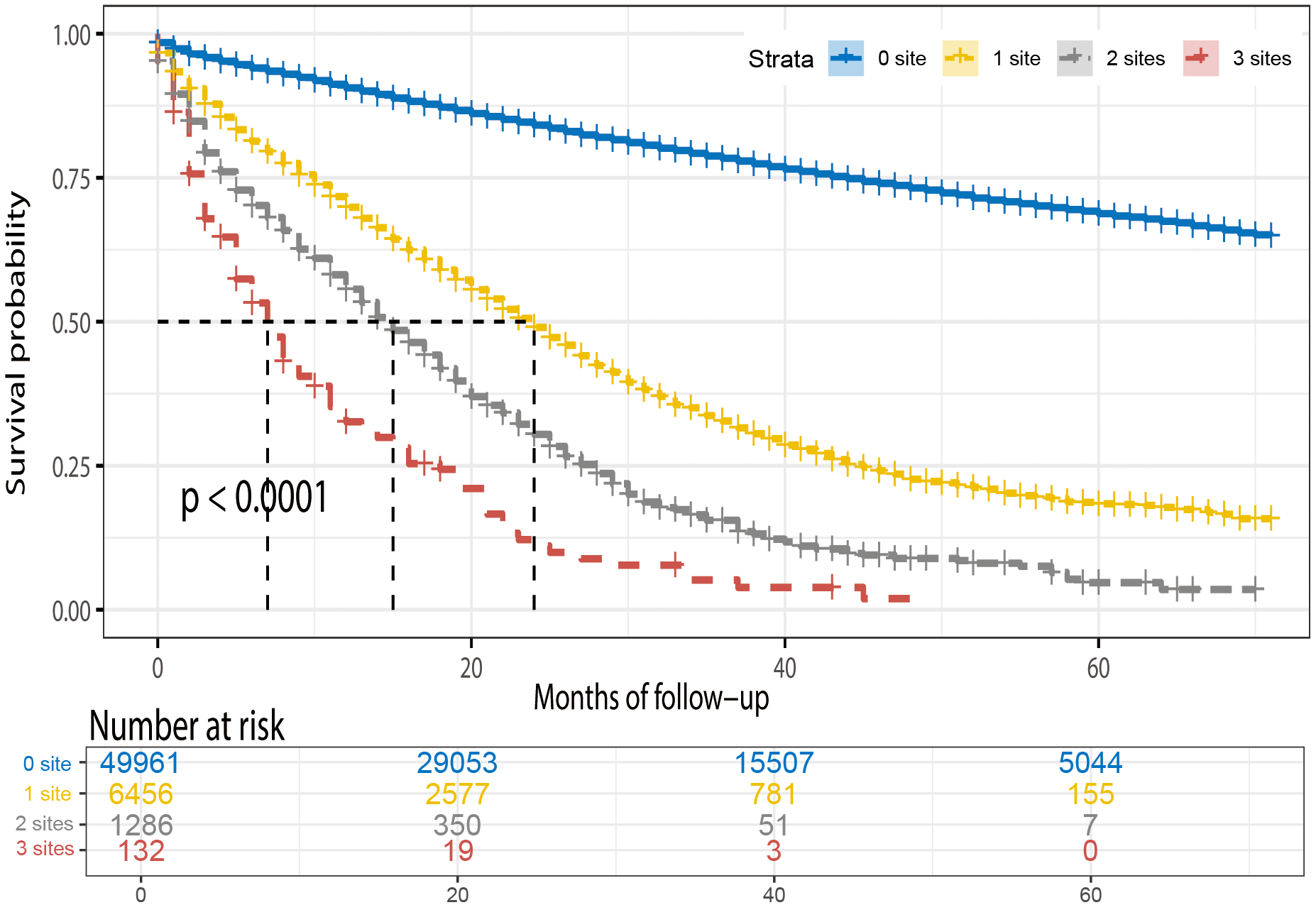

Supplement: Supplementary Figure 3 — Overall survival of patients with newly diagnosed colorectal cancer and extracranial metastases, which was classified by the number of metastatic sites to the bone, lung, or liver. [file Image_3.tif]
