# Supplementary material for: Distant Metastasis Pattern and Prognostic Prediction Model of Colorectal Cancer Patients Based on Big Data Mining
Source: Front Oncol. 2022 Apr 22;12:878805. doi: 10.3389/fonc.2022.878805 (PMC9074728; doi:10.3389/fonc.2022.878805)
Supplement: Supplementary Table 1 — GO and KEGG enrichment analysis of DEGs. [file Table_1.docx]

**Supplement Table 1. GO and KEGG enrichment analysis of DEGs**

| Type | ID | *P*. adjust | Gene Count | Description |
| --- | --- | --- | --- | --- |
| BP | GO:0072376 | 2.36E-15 | 19 | protein activation cascade |
|  | GO:0002526 | 8.97E-14 | 20 | acute inflammatory response |
|  | GO:0019835 | 1.37E-10 | 10 | cytolysis |
|  | GO:0002576 | 2.10E-10 | 14 | platelet degranulation |
|  | GO:0008202 | 5.67E-10 | 19 | steroid metabolic process |
|  | GO:0044281 | 2.92E-09 | 43 | small molecule metabolic process |
|  | GO:0072378 | 3.88E-09 | 8 | blood coagulation, fibrin clot formation |
|  | GO:0006953 | 1.22E-08 | 9 | acute-phase response |
|  | GO:0006956 | 3.17E-08 | 12 | complement activation |
|  | GO:0042730 | 8.98E-08 | 7 | fibrinolysis |
| CC | GO:0072562 | 2.95E-24 | 24 | blood microparticle |
|  | GO:0005576 | 2.35E-19 | 79 | extracellular region |
|  | GO:0044421 | 1.98E-17 | 67 | extracellular region part |
|  | GO:0005615 | 7.65E-16 | 62 | extracellular space |
|  | GO:0070062 | 5.76E-15 | 51 | extracellular exosome |
|  | GO:0005788 | 5.76E-15 | 22 | endoplasmic reticulum lumen |
|  | GO:1903561 | 5.96E-15 | 51 | extracellular vesicle |
|  | GO:0043230 | 5.96E-15 | 51 | extracellular organelle |
|  | GO:0044432 | 1.02E-13 | 38 | endoplasmic reticulum part |
| MF | GO:0004857 | 6.79E-06 | 15 | enzyme inhibitor activity |
|  | GO:0008201 | 1.72E-05 | 10 | heparin binding |
|  | GO:0004866 | 1.72E-05 | 10 | endopeptidase inhibitor activity |
|  | GO:0061135 | 1.72E-05 | 10 | endopeptidase regulator activity |
|  | GO:0030414 | 1.72E-05 | 10 | peptidase inhibitor activity |
|  | GO:0005102 | 2.40E-05 | 28 | signaling receptor binding |
|  | GO:0061134 | 6.69E-05 | 10 | peptidase regulator activity |
|  | GO:0005539 | 6.94E-05 | 10 | glycosaminoglycan binding |
|  | GO:0005496 | 8.27E-05 | 7 | steroid binding |
|  | GO:0004497 | 9.70E-05 | 7 | monooxygenase activity |
| KEGG | hsa04610 | 9.55E-19 | 19 | Complement and coagulation cascades |
|  | hsa00982 | 1.40E-09 | 12 | Drug metabolism - cytochrome P450 |
|  | hsa05204 | 4.59E-09 | 12 | Chemical carcinogenesis |
|  | hsa00830 | 6.19E-09 | 11 | Retinol metabolism |
|  | hsa00980 | 2.04E-08 | 11 | Metabolism of xenobiotics by cytochrome P450 |
|  | hsa05020 | 2.72E-06 | 7 | Prion diseases |
|  | hsa00983 | 6.27E-05 | 8 | Drug metabolism - other enzymes |
|  | hsa00140 | 9.35E-05 | 7 | Steroid hormone biosynthesis |
